# Testosterone imbalance may link depression and increased body weight in premenopausal women

**DOI:** 10.1038/s41398-019-0487-5

**Published:** 2019-06-07

**Authors:** Daniela Stanikova, Rachel G. Zsido, Tobias Luck, Alexander Pabst, Cornelia Enzenbach, Yoon Ju Bae, Joachim Thiery, Uta Ceglarek, Christoph Engel, Kerstin Wirkner, Juraj Stanik, Juergen Kratzsch, Arno Villringer, Steffi G. Riedel-Heller, Julia Sacher

**Affiliations:** 1 Institute of Social Medicine, Occupational Health and Public Health, University of Leipzig, Leipzig, Germany; 2 DIABGENE Laboratory, Institute of Experimental Endocrinology, Biomedical Research Center, Slovak Academy of Sciences, Bratislava, Slovakia; 3 Department of Pediatrics, Medical Faculty at the Comenius University, Bratislava, Slovakia; 4 Department of Neurology, Max Planck Institute for Human Cognitive and Brain Sciences, Leipzig, Germany; 5 Emotion & NeuroimaGinG (EGG) Lab, Max Planck Institute for Human Cognitive and Brain Sciences, Leipzig, Germany; 6 Department of Economic and Social Sciences & Institute of Social Medicine, Rehabilitation Sciences and Healthcare Research (ISRV), University of Applied Sciences Nordhausen, Nordhausen, Germany; 7 LIFE-Leipzig Research Center for Civilization Diseases, University of Leipzig, Leipzig, Germany; 8 Institute for Medical Informatics, Statistics and Epidemiology, University of Leipzig, Leipzig, Germany; 9 Institute of Laboratory Medicine, Clinical Chemistry and Molecular Diagnostics, University of Leipzig, Leipzig, Germany; 10 Center for Pediatric Research Leipzig, University Hospital for Children & Adolescents, University of Leipzig, Leipzig, Germany; 11 Clinic of Cognitive Neurology, University of Leipzig, Leipzig, Germany

**Keywords:** Scientific community, Depression

## Abstract

Accumulating evidence supports a link between depression and being overweight in women. Given previously reported sex differences in fat accumulation and depression prevalence, as well as the likely role of sex hormones in both overweight and mood disorders, we hypothesised that the depression-overweight association may be mediated by sex hormones. To this end, we investigated the association of being overweight with depression, and then considered the role of sex hormones in relation to being overweight and depression in a large population-based cohort. We included a total of 3124 women, 970 premenopausal and 2154 postmenopausal from the LIFE-Adult cohort study in our analyses. We evaluated associations between being overweight (BMI >25 kg/m^2^), sex hormone levels, and depressive symptomatology according to Centre for Epidemiologic Studies Depression (CES-D) scores, and explored mediation of depression in a mediation model. Being overweight was significantly associated with depressive symptoms in premenopausal but not postmenopausal women. Both premenopausal and postmenopausal overweight women had higher free testosterone levels compared with normal weight women. Premenopausal women with depressive symptomatology had higher free testosterone levels compared to women without. We found a significant mediation effect of depressive symptomatology in overweight premenopausal women through free testosterone level. These findings highlight the association between being overweight and depressed, and suggest that high free testosterone levels may play a significant role in depression of overweight premenopausal women. Based on this, pharmacological approaches targeting androgen levels in overweight depressed females, in particular when standard anti-depressive treatments fail, could be of specific clinical relevance.

## Introduction

The worldwide increasing prevalence of obesity and depression are among the leading and most challenging health problems faced today, particularly for women^1–3^. In 2016, 40% of the world’s adult female population was overweight and 15% of women were obese^4^, which is the result of an accelerating rate for population-wide obesity over recent decades^5,6^. Depression prevalence has also increased at an alarming rate. Women have a lifetime risk of major depression nearly twice that of men, approximately 5% of women will develop major depression, and depression is expected to be the second leading cause of disability worldwide by the year 2020^7^. Studies have found that higher rates of both depression and obesity diminish quality of life and increase risk of morbidity and premature mortality^8–10^.

Beyond their independent impact on morbidity and mortality, depression and obesity are highly comorbid, and their co-occurrence is largely associated with adverse health^3^. Several large epidemiologic studies and meta-analyses^11,12^ support an association between obesity and depression, which appears to be stronger in women than in men^13–16^. Moreover, being both overweight and depressed is more prevalent in women than in men, indicating a potential common underlying mechanism. Chronic low-grade inflammation^17^, oxidative stress^18^, metabolic and endocrinologic disturbances^19,20^ have been suggested as possible contributing factors.

Given the observed sex differences in both adipose fat accumulation and depression prevalence, it is likely that sex hormones substantially influence the interplay between obesity and depression. Sex hormones interact with main neurotransmitter systems implicated in the regulation of cognition and affect, including acetylcholine, serotonin, dopamine, and norepinephrine^21,22^. Both androgens and oestrogens play a role in emotional processing, memory, and perception, as well as the interpretation of sensory information in the hypothalamus and hippocampus^23^, supporting a significant role in the etiopathogenesis of major depressive disorder. Evidence from several lines of research link hormone fluctuations to increased risk of developing a major depressive episode. Firstly, vulnerability for depression in women significantly increases in puberty and declines after menopause^24^. Secondly, postpartum and perimenopause phases and the immediate period after a surgically induced menopause (all of which are characterised by substantial changes in sex hormone levels—particularly oestradiol) are associated with a significant increase in the risk of depression in women with or without a previous history of depressive symptoms^25,26^. Finally, studies in clinical populations found associations between sex hormones, such as oestradiol and testosterone, and depression. Sustained low oestradiol levels have been observed in women with major depressive disorder^27,28^, as well as clinical recovery from depression during the postpartum period, perimenopause, and postmenopause following restoration of stable oestradiol levels^28^. Antidepressant effects of oestrogen were observed in several animal studies^29,30^ and have been shown to be dose and receptor-dependent^31^. While alterations in testosterone levels are largely accepted to influence the pathogenesis of depression in men^32–35^, recent findings suggest that testosterone may play a critical role in depression susceptibility in women as well, although the findings are mixed^36–38^. While the concentration of testosterone in women is ten times lower than in men^39^, it has been shown that women are more sensitive to it^40^. In a study of women with polycystic ovarian syndrome, a state of hyperandrogenism, a four-fold risk increase for depression independent of body mass index (BMI) was found^41^. Several animal studies have also indicated an anti-depressant effect of testosterone^42,43^ and this effect appears to be dose dependent^39^

Propensity towards development of obesity differs between the sexes and has been linked to endogenous changes in sex hormones: Increased body fat mass in women was shown to be associated with alterations of sex hormone levels^44^. Human adipose tissue represents an intracrine source of androgen synthesis in females, contributing to changes in circulating sex hormone levels and dysregulation of the hypothalamic-pituitary-gonadal axis^45^. Unlike in men, serum androgens positively correlate with increased BMI in women^46^. Moreover, it has also been shown that the hormonal abnormalities are partly or wholly reversible with weight loss, indicating a causality of this relationship ^47^.

**Fig. 1:**
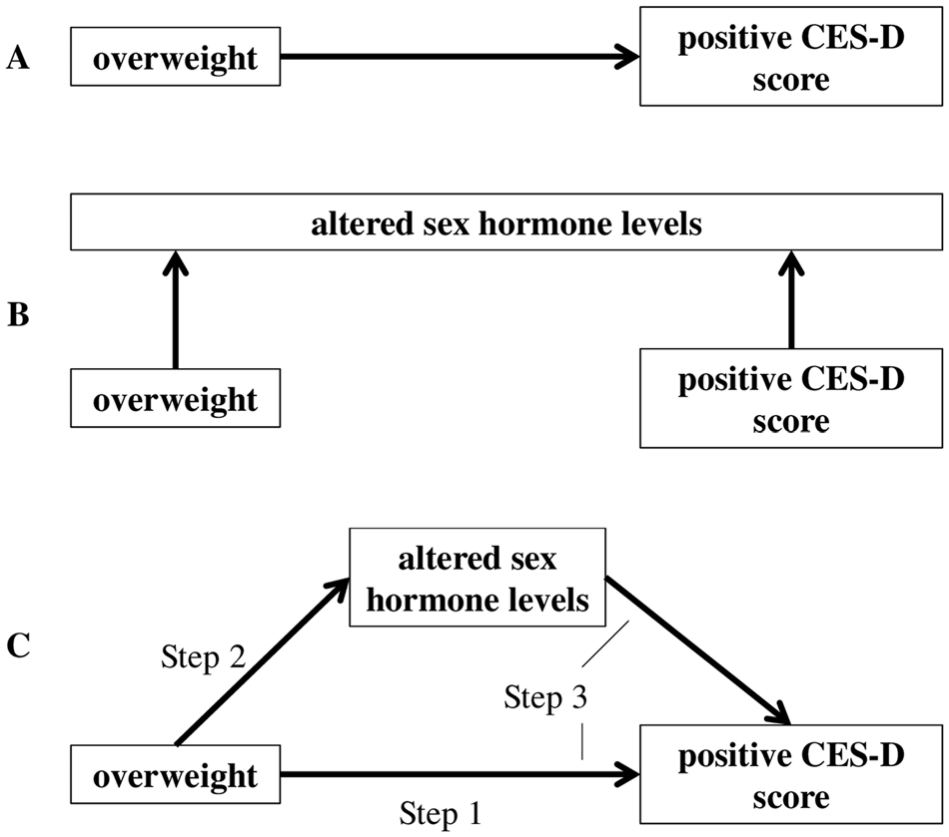
Hypothesis. We tested the association between **a** depressive symptomatology and overweight, **b** association between overweight and depressive symptomatology with sex hormone levels, and **c** whether the association between depression and overweight might be mediated through altered sex hormone levels associated with increased BMI. Step 1 in **c** shows an association between overweight (≥25 kg/m²) and depressive symptomatology (CES-D ≥ 23 points). Step 2 in **c** shows an association between overweight and sex hormone level and Step 3 in **c** shows an association between depressive symptomatology, overweight, and sex hormone level

What remains uncertain is whether and how these BMI-associated sex hormone alterations may impact mood and depressive symptoms. We hypothesise that all three conditions —increased BMI, alterations in sex hormone levels, and depression are strongly interconnected. More specifically, these hormonal changes may actually mediate depression in overweight women. Here, we aim to replicate the association between increased body weight and depressive symptoms in a well-powered (*n* > 3000 women) cohort and subsequently determine whether the association between being overweight and depressed correlates with sex hormone levels. Finally, we will investigate whether sex hormone levels mediate the association between being overweight and depressed (Fig. 1).

## Patients and methods

### Patients

Data presented in this study were obtained from the Leipzig Research Centre for Civilisation Diseases (LIFE) Adult Study, a population-based cohort study of adults from the eastern part of Germany (Leipzig) with over 10,000 randomly selected participants. The objective of the LIFE Adult Study is to investigate prevalence, early onset markers, genetic predispositions, and the role of lifestyle factors in major diseases of civilisation, including metabolic diseases and depression. Written informed consent was obtained from all participants. The study was approved by the responsible institutional ethics board of the Medical Faculty of the University of Leipzig (PV 2016-274-04). The data privacy and safety concept of the study was endorsed by the responsible data protection officer^48^.

### Medical history, medications

Participants were asked about medical diagnoses previously confirmed by a physician. Seventy common medical diagnoses were included in the interview. Data on menstrual cycle (number of days/months/years since the last menstrual period), parity, history of bilateral oophorectomy and hysterectomy, as well as past or present use of contraception pills or hormonal replacement therapy were included. Data on all other medications taken within the past seven days before the study day were gathered. Medications were identified by barcodes^48^, following The Anatomical Therapeutic Chemical Classification System (ATC) classification.

### Anthropometric measurements

Anthropometric measurements were assessed by trained study personnel according to standardised protocols. Body weight was measured with an electronic scale (SECA 701, Seca Gmbh & Co KG) with a precision of 0.01 kg; body height was assessed using a stadiometer (SECA 240) to the nearest 0.1 cm. BMI was calculated as weight in kilograms divided by the square of the body height in metres. Underweight was assessed as BMI < 18.5 kg/m^2^, normal weight as BMI > 18.5 and <25 kg/m^2^, overweight as BMI > 25 kg/m^2^ (pre-obesity BMI > 25 and 29.9 kg/m^2^ and obesity >30 kg/m^2^)^11^.

### Depression screening

Current (within the last week) depressive symptoms were evaluated by a self-report questionnaire, using the German version of the Centre for Epidemiological Studies Depression Scale (CES-D)—“Die Allgemeine Depressionsskala-Langform” (ADS-L). This screening tool is a reliable and valid instrument for detecting depressive symptoms with broad applicability in the general population. The CES-D (ADS-L) consists of 20 items and ranges from 0 to 60, higher scores indicating more depressive symptoms^49^. A score ≥23 is suggested to indicate clinically relevant depressive symptoms^50^.

### Hormonal analyses

Blood samples were collected between 7:00 and 10:30 a.m. in a fasting state. Analyses of sex hormones (sex hormone-binding globulin, oestradiol, total testosterone) as well as albumin (for calculation of free testosterone) were performed on fresh biospecimens on the day of sample collection. Biochemical analysis was performed by fully the automated Cobas system (Roche, Mannheim). Intra-assay and inter-assay coefficients of variation were given exemplarily by 100 subsequent analyses during the time of recruitment study participants: The results of sex hormone-binding globulin were <3.4% for the range between 25.4 and 54.2 nmol/L, the results of total testosterone were <4.9% for the range between 3.73 and 19.07 nmol/L, and the results for oestradiol were <4.3% for the range between 387–2052 pmol/L. Free and bioavailable testosterone were calculated using Vermeulen’s formula^51^, and free oestradiol using the spreadsheet method^52^. We used the classification of the menopausal status according to the Breast Cancer Surveillance Consortium^53^ as follows: premenopausal (0–6 months since the last menstrual period (LMP) or <45 years), perimenopausal (6–12 months since LMP), postmenopausal (>12 months since LMP or ≥55 years or bilateral oophorectomy).

### Exclusion criteria

**Fig. 2:**
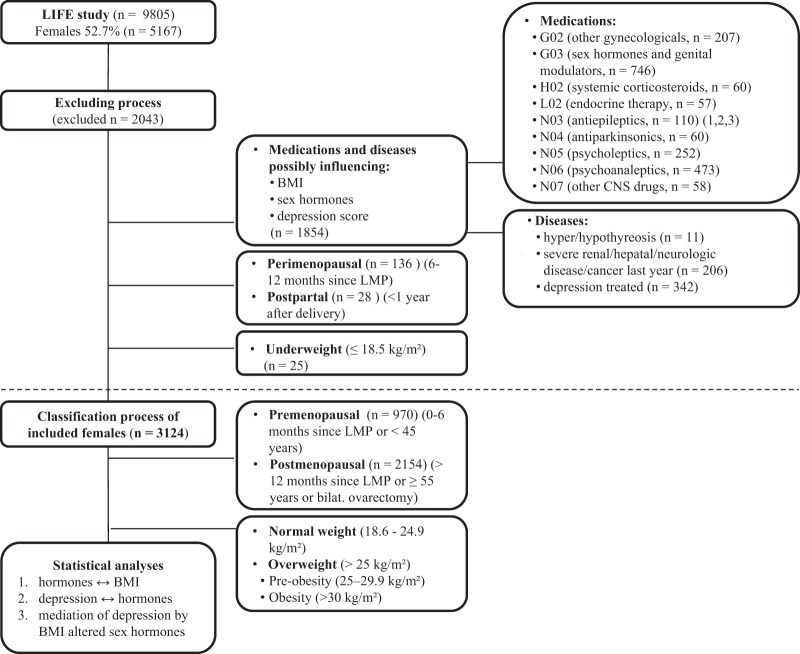
Study design. Exclusion criteria and classification of females. G02, G03, H02, L02, N03-N07—ATC groups of therapeutics. BMI, body mass index; LMP, last menstrual period

The total sample included 5167 women with a mean age of 56.6 years (range 18.8–82.1 years) and mean BMI of 27.0 kg/m^2^ (range 16.1–56.7 kg/m^2^). Most of the women were born in Germany (95%) and had a middle (52.8%) or high (27.4%) educational status. Seventy percent of them were married or living with a partner. Due to the possible impact on either sex hormones, BMI or depression score, we excluded all women who were using external hormones or CNS drugs (with the exception of analgetics and local anaesthetics), women with hypothyreosis and hyperthyreosis, severe chronic renal, hepatic, or neurological diseases, had cancer within the last year, and women who had already been diagnosed and treated (pharmacologically or non-pharmacologically) for depression within the last year. We also excluded perimenopausal and postpartum (1 year after delivery) women, as both periods are related to fluctuations in sex hormones, BMI changes, and increased onset and prevalence of depressive and anxiety disorders^24^. Detailed exclusion criteria including ATC categories of excluded medications are provided in Fig. 2.

### Statistical analyses

The analyses were carried out separately for the groups of premenopausal and postmenopausal women. Categories for being overweight (BMI > 25 kg/m^2^) and depressed (CES-D ≥23 points) were used as discontinuous variables similarly to other studies^11,54^. Comparison between groups was tested using *t*-tests for continuous data and by chi-square tests for binary data. Values for the cohort description are given as mean ± SD. To assess the potential mediating effect of sex hormone levels on the relationship between being overweight and depressed, we tested the mediation hypothesis as described by Baron and Kenny^54^. Briefly, testing mediation require three regression models in which the following conditions are met: (1) The predictor variable is significantly correlated with the outcome variable when ignoring the mediator variable, (2) the predictor variable is significantly correlated with the mediator variable and (3) the effect of the predictor variable on the outcome variable after controlling for the mediator variable should be greatly reduced or become non-significant (Fig. 1). For the third step, full mediation occurs if the effect of the predictor variable on the outcome variable becomes non-significant after controlling for the mediator, and a partial mediation occurs if the effect becomes significantly reduced but remains significant. Partial mediation means that the mediating variable accounts for some (but not all) of the relationship between the independent and dependent variable. In our model, the predictor variable was being overweight (BMI > 25 kg/m^2^) and the outcome variable was being depressed (CES-D ≥23 points), both of which were defined as binary (present = 0, not present = 1). The mediator variable (free testosterone levels) was entered as a continuous variable. Enter binary logistic regression was used for all three models. The significance threshold for all statistical analyses was set at <0.05, and all analyses were performed using SPSS version 25.0 (IBM, NY, USA).

## Results

### Subjects included

The study sample included 3124 women of whom 970 (31.0%) were premenopausal (45 ± 6.6 years) and 2154 (68.9%) were postmenopausal (64.2 ± 8.0 years). In the group of premenopausal women, 54.3% were of normal weight (mean BMI 22.1 ± 1.6 kg/m^2^) and 45.6% were overweight (28.4% were pre-obese, mean BMI 27.1 ± 1.4 kg/m^2^ and 17.2% were obese, mean BMI 35.1 ± 5.0 kg/m^2^). In the group of postmenopausal women, 32% were of normal weight (mean BMI 22.8 ± 1.5 kg/m^2^) and 67.7% were overweight (38.2% were pre-obese, mean BMI 27.4 ± 1.4 kg/m^2^ and 29.5% were obese, mean BMI 34.1 ± 4.1 kg/m^2^). Seven hundred twenty-two women (73.6%) in the premenopausal group and 1908 (88.0%) in the postmenopausal group were parous.

#### Association between being overweight and depression

In the premenopausal group, we found significantly higher CES-D scores in overweight women compared with women of normal weight (11.2 ± 7.7 vs. 10.0 ± 7.4, *p* = 0.01). Overweight premenopausal women also had a significantly higher prevalence of depressive symptomatology compared to women of normal weight (10.5% vs. 6.6%, *p* = 0.042) with the OR = 1.65 (CI = 1.032–2.651, *p* = 0.036). In the postmenopausal group, we observed no significant differences in overweight versus women of normal weight in depressive symptomatology (6.5% vs. 6.1%, *p* = 0.841; OR = 1.05, CI = 0.709–1.571, *p* = 0.796).

Among other factors associated with increased BMI, we did not find a significant association of depressive symptomatology with either waist circumference (89.0 ± 12.7 cm in group without vs. 87.4 ± 12.9 cm in group with depressive symptomatology, *p* = 0.296 in premenopausal and 95.3 ± 12.6 vs. 94.1 ± 12.5 cm, *p* = 0.339 in postmenopausal women) waist-hip ratio (0.8 ± 0.1 vs. 0.8 ± 0.1, *p* = 0.217 in premenopausal and 0.9 ± 0.1 vs. 0.9 ± 0.1, *p* = 0.172 in postmenopausal women) or hs-CRP (1.4 ± 1.4 vs. 1.9 ± 2.7 mg/L, *p* = 0.627 in premenopausal and 2.1 ± 1.8 vs. 1.6 ± 1.5 mg/L, *p* = 0.133).

We also observed no significant effect of parity on BMI (25.3 ± 5.2 kg/m^2^ for nonparous vs. 26.0 ± 5.5 kg/m^2^ for parous, *p* = 0.069 in the premenopausal group, and 28.2 ± 4.9 kg/m^2^ for nonparous vs. 27.8 ± 5.2 kg/m^2^ for parous, *p* = 0.371 in the postmenopausal group, respectively), prevalence of being overweight (41.3% in nonparous vs. 47.2% in parous, OR = 1.27, CI = 0.95–1.69, *p* = 0.124 in the premenopausal, and 71.7% vs. 66.7%, OR = 0.79, CI = 0.59–1.05, *p* = 0.120 in the postmenopausal group), or prevalence of depressive symptomatology (9.0% in nonparous vs. 8.2% in parous, OR = 0.91, CI = 0.54–1.52, *p* = 0.689 in the premenopausal, and 3.9% vs. 6.7%, OR = 1.74, CI = 0.87–3.49, *p* = 0.146 in the postmenopausal group, respectively).

#### Associations of being overweight and depression with sex hormone levels

##### Associations of being overweight with sex hormone levels

**Fig. 3:**
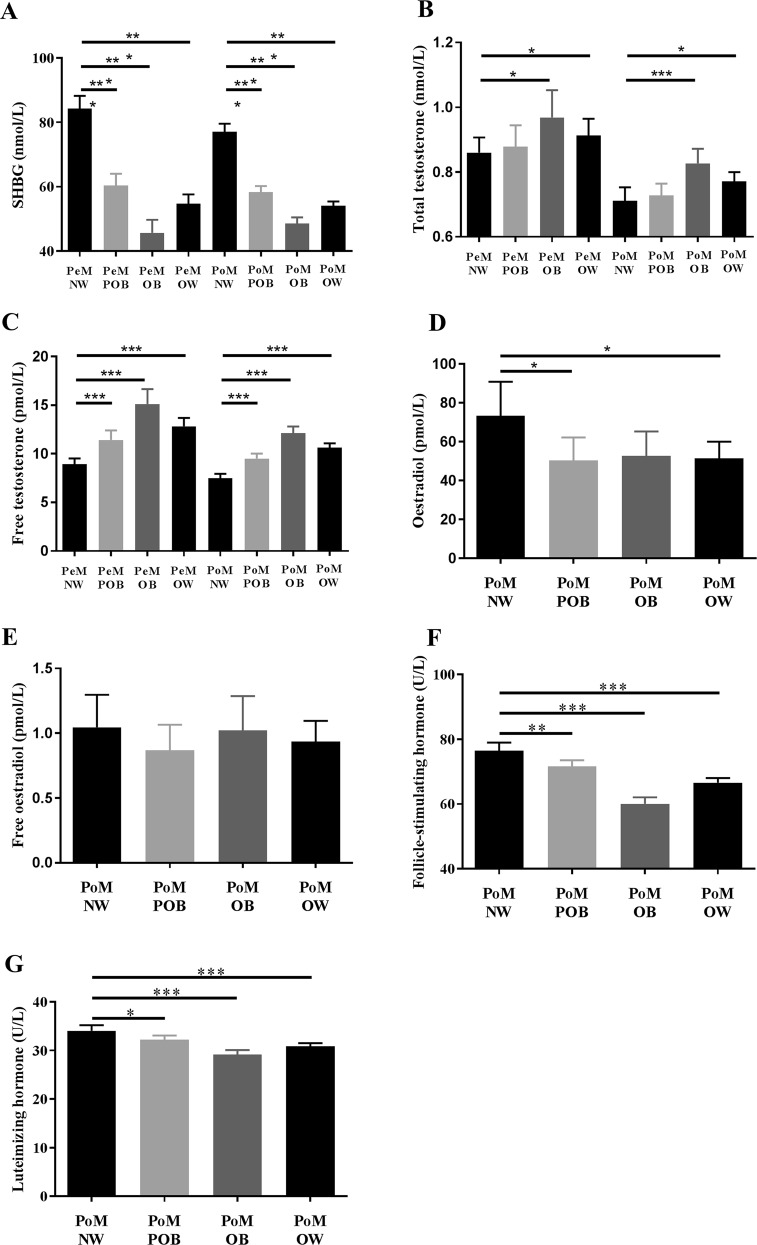
Levels of selected sex hormones in various BMI groups in premenopausal and postmenopausal females. **a** Sex hormone-binding globulin, **b** total testosterone, **c** free testosterone, **d** oestradiol, **e** free oestradiol, **f** follicle-stimulating hormone, **g** luteinizing hormone. Values are displayed as mean + confidential intervals of the mean. PeM, premenopausal females; PoM, postmenopausal females; NW, normal weight; POB, pre-obese (>25 <30 kg/m^2^), OB, obese (>30 kg/m^2^), OW, overweight (>25 kg/m^2^). Significant differences *p ≤ 0.05, **p < 0.01, and ***p < 0.001

Compared with individuals of normal weight, overweight (including both subgroups of pre-obese and obese) women had lower levels of sex hormone binding globulin (54.8 ± 27.5 vs. 84.2 ± 41.8 nmol/L, *p* < 0.001 in premenopause, and 54.1 ± 23.5 vs. 73.1 ± 25.5 nmol/L, *p* < 0.001 in postmenopause), higher total testosterone level (12.8 ± 8.3 vs. 8.9 ± 6.1 pmol/l, *p* < 0.001 in premenopause, 10.6 ± 7.5 vs. 7.5 ± 5.6 pmol/L, *p* < 0.001 in postmenopause) and free testosterone level (for details see Fig. 3).

Because of fluctuations of oestradiol levels during the menstruation cycle in premenopausal women, the association of BMI with oestradiol levels in premenopausal was assessed by Pearson’s partial correlations controlled for age and phase of menstrual cycle. No significant association of BMI with serum oestradiol levels (*R* = −0.022, *p* = 0.536) or free oestradiol levels (*R* = 0.055, *p* = 0.120) in premenopausal women was found.

In postmenopausal overweight women, we found significantly lower oestradiol levels (73.2 ± 211.1 L^−1^, vs. 51.4 ± 149.4 pmol/L, *p* = 0.014) compared to women of normal weight. We also found significantly lower levels of luteinizing hormone (30.8 ± 11.1 vs. 34.1 ± 13.7 U/L, *p* < 0.001) and follicle-stimulating hormone (66.5 ± 24.8 vs. 76.4 ± 30.1 U/L, *p* < 0.001) in overweight women compared to women of normal weight.

##### Association of depression with sex hormone levels

Premenopausal women who reported depressive symptoms had significantly higher free testosterone levels compared to women who did not report being depressed (12.9 ± 9.1 vs. 10.4 ± 7.0 nmol/L, *p* = 0.032).

**Fig. 4:**
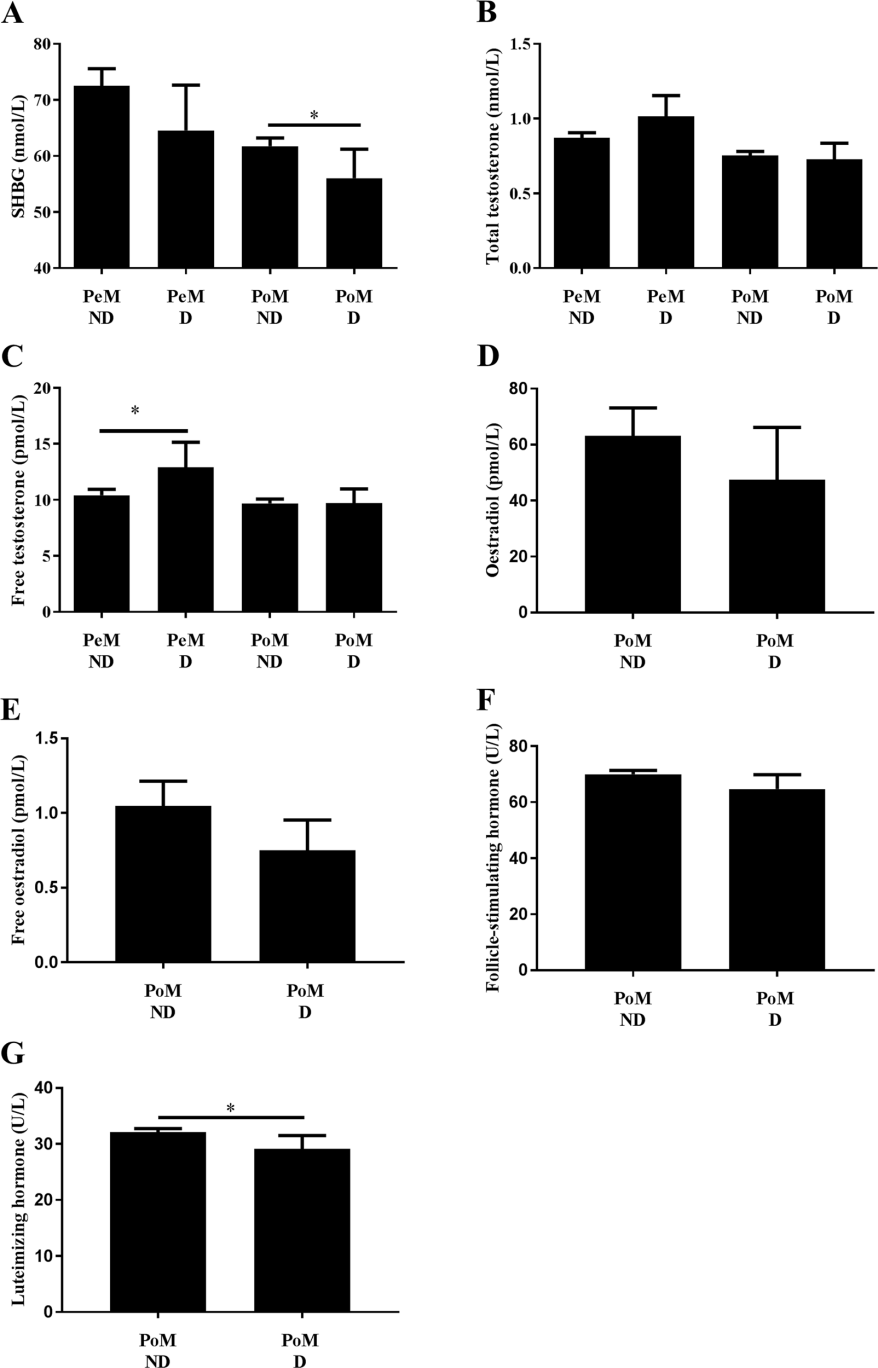
Levels of selected sex hormones in premenopausal and postmenopausal women with and without presence of depressive symptomatology. **a** Sex hormone-binding globulin, **b** total testosterone, **c** free testosterone, **d** oestradiol, **e** free oestradiol, **f** follicle-stimulating hormone, **g** luteinizing hormone. Values are displayed as mean + confidential intervals of the mean. PeM, premenopausal females; PoM, postmenopausal females; D, depressed/presence of depressive symptomatology (CES-D ≥ 23 points), ND, non-depressed/without presence of depressive symptomatology. Significant differences *p ≤ 0.05, **p < 0.01, ***p < 0.001

Postmenopausal women with positive depressive symptomatology had significantly lower levels of sex hormone-binding globulin (56.0 ± 26.7 vs. 61.7 ± 28.0 nmol/L, *p* = 0.044) as well as luteinizing hormone (29.1 ± 12.4 vs. 32.1 ± 12.3 U/L, *p* = 0.016) (Fig. 4).

#### Sex hormone levels and the association of being overweight and depression

**Fig. 5:**
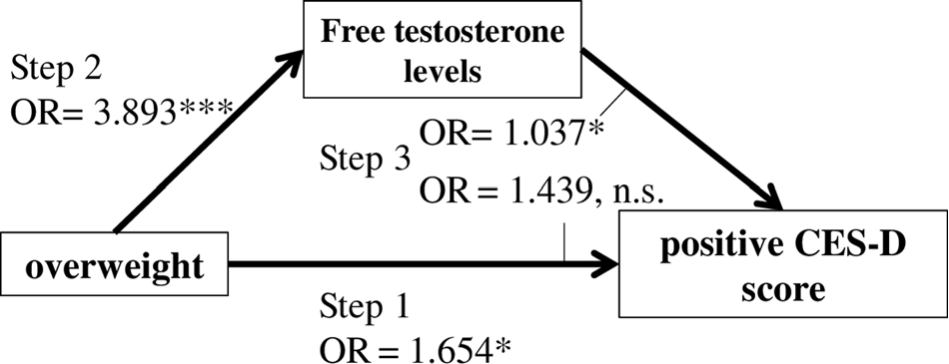
Mediation model in premenopausal women. In Step 1, we found a significant positive association between being overweight and depressive symptomatology. In Step 2, we found a significant association between being overweight and free testosterone level. In Step 3, we found a significant association between free testosterone level, but not overweight, with depressive symptomatology. This means a complete mediation of association between being overweight and depressive symptomatology by free testosterone level

Subsequently; we explored a potential mediation of depression by sex hormone levels in overweight women and found a significant mediation effect in the premenopausal group only. In postmenopausal women, we did not find any significant association of being overweight and depression. In premenopausal women, we then used the mediation model to examine the respective associations (Fig. 1c): In the first step of the model, we report a significant association (OR = 1.65, CI = 1.032–2.651, *p* = 0.036) of being overweight and depressive symptomatology. We also observed a significant association of being overweight and free testosterone levels in the second step (OR = 3.89, CI = 2.881–4.905, *p* < 0.001). In the third step, we found that free testosterone (OR = 1.03, CI = 1.005–1.070, *p* = 0.022), but not being overweight (OR = 1.43, CI = 0.847–2.443, *p* = 0.178), was significantly associated with depression (Fig. 5).

## Discussion

Here, we present empirical data from a large population-based cohort study for a link between being overweight, being depressed, and sex hormone levels before menopause. Given the rising rates of obesity and depression worldwide, recent findings highlight that the often-reported relationship between obesity and depression may be of a sex-specific nature^55,56^, and of particular relevance in women. This important observation still lacks evidence from large population-based cohort studies for validation and for framing essential questions on possible mechanisms underlying this relationship.

In this study, we first replicated a positive association between being overweight and being depressed in our sample of 970 premenopausal women, previously reported in a smaller sample size^55^. We additionally provide novel evidence for a link between sex hormone levels, depression, and obesity in women during the reproductive years. We found an association between premenopausal free testosterone levels and being overweight, as well as with depression. Further exploratory mediation analysis suggests that depressive symptomatology in overweight premenopausal women may be mediated via free testosterone levels.

### Association of depression and being overweight

Several large epidemiological studies and a meta-analysis report a positive association between being overweight and depression^11,57^, and this association appears to be more prevalent in women than in men^11^. Increased total and regional percentage fat body mass have been shown to be significantly associated with depression in both men and women in a recent study conducted by Li and colleagues^58^. In one of the largest studies to date (over 177,000 individuals)^12^, a significantly higher prevalence of depressive symptomatology was found in overweight (both pre-obese and obese) women.

However, not all studies report consistent evidence for an association between depression and being overweight^59^. These conflicting reports could be explained by differences in sample characteristics and methods (e.g., a variety in age range, sample sizes, methods of assessments for depression, or anthropometric measurements). While some studies claim that the association of depression and obesity might be bilateral^11^, Geoffroy et al.^57^ argue that obesity predicts subsequent depressive symptoms, but not vice-versa, as supported by data from approximately nineteen thousand individuals. In that study, they found that obesity predicted depressive symptoms only in females, who had been recruited at birth and were re-assessed longitudinally for 50 years. Similarly, we replicate the association of depression with increased body weight in premenopausal women (45 ± 6.6 years) but not in postmenopausal women (64.2 ± 8.0 years). This finding is in line with a phenomenon that is often referred to as the “Jolly Fat” hypothesis (a lower prevalence of depression associated with a moderately increased in BMI in older individuals) and has been supported by previous studies, both for overweight men and overweight women in later life^60^.

While we did observe a strong relationship between depressive symptomatology and BMI in the premenopausal group, there was no association between CES-D scores and either waist hip ratio or waist circumference. This may indicate that it is not adipose tissue distribution in premenopausal women inducing depression but rather the general increase in overall body weight exceeding a normal range. This speculation could be partially supported by the fact that peripheral type of obesity is typically predominant in premenopausal women, rather than the more abdominal/visceral fat accumulation pattern observed later in life for women. Thus, measures such as waist hip ratio and waist circumference may not yet be able to capture the obesity-associated changes in mood in this younger cohort.

Unlike other studies^61,62^, we also did not prove a significant association of depressive symptomatology with hs-CRP, another factor associated with obesity. However, our results might be influenced by the fact that hs-CRP values were only available in a minor part of women, and predominantly healthy participants.

### Association of sex hormone levels and being overweight

We found higher total and free testosterone levels and lower sex hormone-binding globulin levels in overweight premenopausal and postmenopausal women compared with women of normal weight. These findings are in concordance with several other reports of an association of increased BMI with a more androgenic profile (diminished sex hormone-binding globulin and elevated free testosterone levels) in women, independent of menopausal status^63^: Obese women, particularly those with abdominal fat accumulation, tend to develop a condition of functional hyperandrogenism. This is because female adipose tissue is an intracrine source of androgen synthesis, as preadipocytes convert androstenedione to testosterone and thus actively contribute to androgen synthesis. This is most likely catalysed by 17-HSD5. Circulating levels of dehydroepiandrosterone sulphate (DHEAS), a crucial androgen precursor, correlate positively with truncal fat in women, whereas there is an inverse correlation in men^64^. In line with these reports, we found increased testosterone levels with increased BMI in postmenopausal women. However, the average levels of testosterone were still within reference ranges (Fig. S1).

We do not report a significant correlation between serum oestradiol levels and increased BMI in premenopausal women. This finding is in concordance with other studies which have shown that, although oestradiol production in adipose tissue positively correlates with BMI^39^, it does not seem to significantly influence oestradiol levels in serum^65^.

We found significantly lower oestradiol levels in postmenopausal overweight women compared with postmenopausal women of normal weight. This finding corresponds with significantly lower levels of luteinizing hormone and follicle-stimulating hormone in overweight postmenopausal group found also by other studies^66,67^.

#### Association of sex hormones and depression

Phases of steep hormonal fluctuations, mostly during perimenopause and the postpartum period^26^, or conditions of sex hormone imbalance (such as hyperandrogenism in e.g., polycystic ovarian syndrome) are undoubtedly associated with an increased risk of depression in women^41,68^. In our study, we excluded women meeting these criteria to investigate whether sex hormones levels are associated with depression outside these high-risk situations. In line with previous studies, we found significantly higher levels of free testosterone in premenopausal women with depressive symptomatology compared with premenopausal women who do not report any depressive symptomatology.

Several studies have reported an association of supraphysiological testosterone levels in women and depression^69^. Interestingly, higher testosterone levels have also been reported in women suffering from postpartum “baby blues”^70^. Furthermore, elevated testosterone levels, together with hypercortisolemia^69^, have been observed in women with severe depression, suggesting an overstimulation of the adrenal glands by hyperactivity of the hypothalamic-pituitary-adrenal axis, which is closely intertwined with gonadal steroid hormone synthesis and activity^71^. However, not all studies report a consistent association between depression and testosterone level in women^72^. These inconsistencies could in part be driven by the different designs of these studies, varying exclusion criteria used, as well as the differences in classification of women according to the menopausal status and evaluating these associations for each group separately.

We did find lower sex hormone-binding globulin levels in postmenopausal women with depressive symptomatology compared with healthy postmenopausal women. We are aware of only of one study to date where such an association of sex hormone-binding globulin levels and depressive symptomatology was observed in early postmenopausal women (<10 years after menopause)^73^. In a rodent model, Caldwell and colleagues^74^ demonstrated internalisation of sex hormone-binding globulin in the hypothalamus, which might indicate a potential role of sex hormone-binding globulin in the pathogenesis of depression. Future epidemiologic studies are needed to confirm our findings of this association in postmenopausal women.

The negative association that we observed in postmenopausal women between depression and luteinizing hormone has also been described in previous studies^75,76^. In the study of Altman^75^, mean plasma luteinizing hormone concentration in postmenopausal women suffering from unipolar depressive disorder was 33% lower than that of normal postmenopausal women.

### Role of sex hormones in the association between being overweight and depressed

To the best of our knowledge, this is the first study to examine the role of sex hormones as a potential mediator in the interplay between obesity and depression in a large population-based cohort. Our findings suggest that the relationship between increased body weight and depression could be mediated by free testosterone levels. Several lines of argument point towards the conclusion that the correlation of testosterone levels with being overweight and being depressed may not be purely coincidental: (1) Hormonal transition periods, particularly functional hyperandrogenic states, have been associated with increased rates of depression. Testosterone levels have been shown to significantly increase serotonin transporter binding^77^, thus suggesting that testosterone triggers a depressed mood by inducing synaptic serotonergic depletion via increased neuronal re-uptake. (2) Studies in patients with major depressive disorder provide evidence of a normalisation of increased testosterone levels by standard antidepressant treatment^78^. (3) Severity of obesity seems to be reflected by the extent of sex hormone alterations^79^ as well as by severity of depression^11^. Moreover, the sex hormonal changes associated with increased body weight accumulation seem to be reversible with subsequent weight loss, which suggests body weight to causally influence sex hormone levels.

These lines of evidence, as well as our data, support an essential role of testosterone in mediating depression in overweight premenopausal women. However, other factors should be considered. In addition to more characteristic risk factors for depression in women (such as age, fluctuating sex hormone levels, and failures in interpersonal relationships)^80,81^, specific psychosocial pressures associated with increased body weight (such as weight discrimination, low self-confidence, and body-dissatisfaction) may trigger or contribute to a depressed phenotype, specifically in overweight women during their reproductive years^82^. Within such a framework, a sex hormone imbalance, associated and potentially exacerbated by abdominal fat tissue accumulation, could provide a “vulnerable terrain” for developing depression^80^.

Hormonal changes according to the BMI were similar in premenopausal and postmenopausal women (Fig. 3), however, we did not find significant associations of depression symptomatology with free testosterone levels or being overweight in the postmenopausal group. This indicates that additional factors in postmenopausal group could play a role, e.g., hormonal sensitivity, absolute hormonal levels that differed compared to premenopausal group, and “Jolly Fat” hypothesis—lower prevalence of depression in older overweight women^60^.

### Strengths and limitations

To the best of our knowledge, our dataset, which explored associations between BMI, sex hormones, and depression in more than 3000 women, is the largest to date. The adherence to the strict exclusion criteria employed in the aim to show “clear associations” represents a second major strength of this study. Further, anthropometric measurements were acquired by trained and experienced study personnel, rather than based on self-reports, which has been shown to limit data interpretation in previous work^83^. We acknowledge that additional information on menstrual phase would have been valuable for the interpretation of oestradiol levels in premenopausal women. However, as the blood-draw could not be performed at the same menstrual cycle phase in all subjects in this large cohort, we addressed this concern by standardised use of adjusted levels of oestradiol for one (follicular) phase in all statistical analyses. While this standardised approach does not reflect individual phase, distributions of menstruation phases were similar in all BMI groups, which suggests that our findings are not substantially driven by menstrual cycle phase only. We further acknowledge that we can only report correlation and cannot infer causation because of the cross-sectional nature of our data. While the mediation analysis provides a snapshot of the potential relationship and directions between variables, this approach is not without limitations. Longitudinal studies, as well as interventional designs are needed to ultimately clarify causality and direction in the relationship of sex hormones, obesity, and depression. By integrating sex hormone data with depression scores and thorough anthropometric measures in a large population-based sample of more than 3000 women, we aim to provide the critical evidence-based model for the role of sex hormones in the relationship of obesity and depression, which needs validation in further studies.

In conclusion, we have demonstrated how sex hormone levels can be integrated into a risk model for depression in overweight postmenopausal women by providing evidence to suggest that higher free testosterone level may be a mediating factor of depression in overweight premenopausal women. As hyperandrogenism associated with increased abdominal fat accumulation has been shown to be reversible with weight loss^84–86^, our findings emphasise the importance of maintaining a healthy body weight for women in early adult and midlife for effective treatment, and possibly prevention of depression in later life. Based on this insight, pharmacological approaches targeting androgen levels in overweight depressed females, in particular when standard anti-depressive treatments fail, could be of specific clinical relevance.

## Supplementary information

S1_Fig
