# Supplementary figures and images for: Testosterone imbalance may link depression and increased body weight in premenopausal women
Source: Transl Psychiatry. 2019 Jun 7;9:160. doi: 10.1038/s41398-019-0487-5 (PMC6555814; doi:10.1038/s41398-019-0487-5)

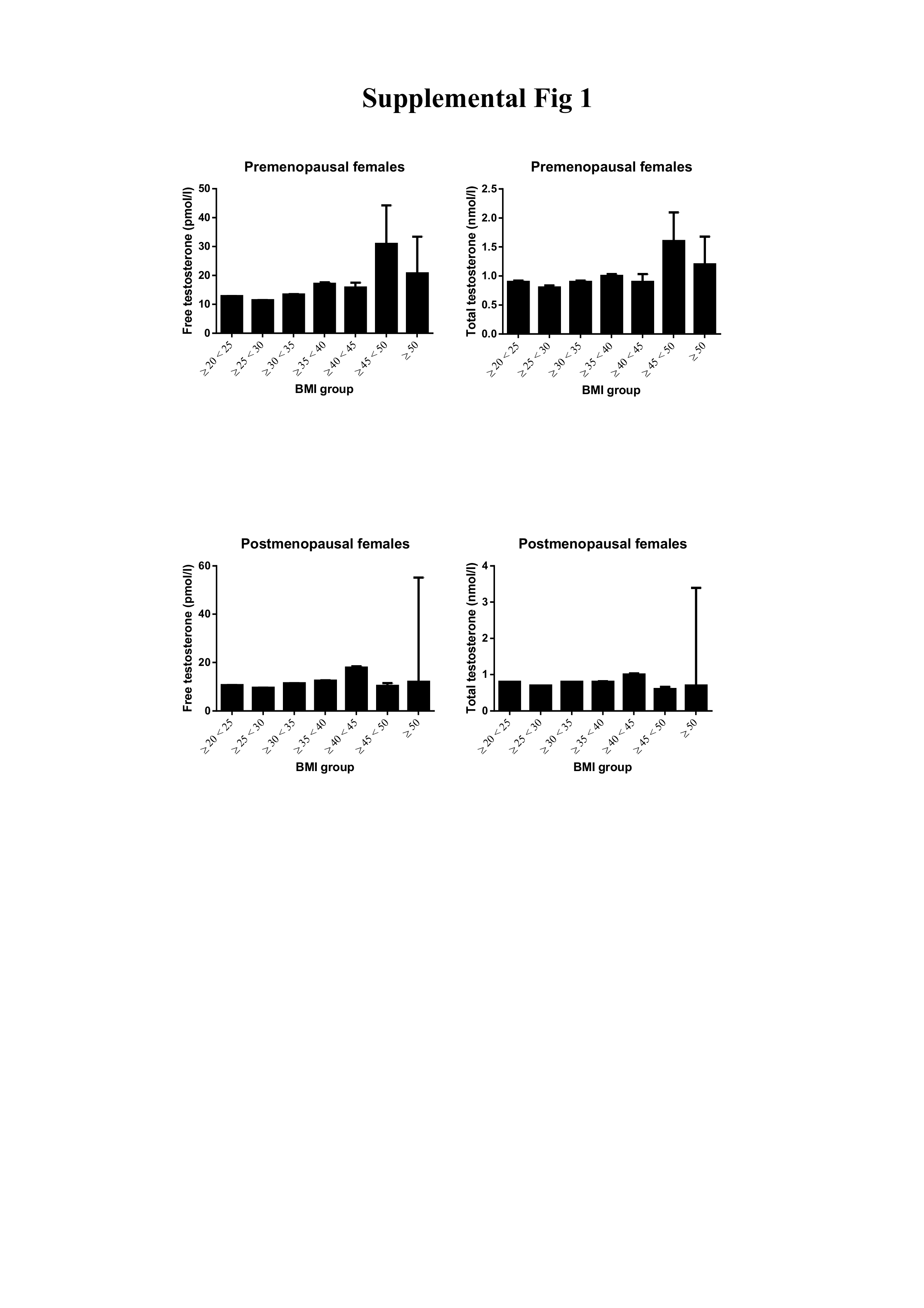

Supplement: Supplementary file 1 — S1_Fig [file 41398_2019_487_MOESM1_ESM.tif]
